# Transactions of the Alumni Society of the University of New York

**Published:** 1842-12

**Authors:** 


TRANSACTIONS OF THE ALUMNI SOCIETY OF THE UNIVERSITY OF NEW YORK.

The alumni of the College of Physicians and Surgeons of the State of New York, have issued a pamphlet of sixteen closely and neatly printed pages, giving an account of their first annual meeting and of their plan for future operations. This report embraces, for so small a publication, a great variety of exceedingly interesting matter, as will be seen by an inspection of the list of articles. The first paper is on the statistics of Fort Fairfield, Maine, by Dr. Coolidge of the U. S. A., and contains memoranda of the weather, facts and discussions concerning the pathology and treatment of delirium tremens, and valuable statistics relative to variola. The second article is an ingenious one, being an attempt at an explanation of the difference in size of the male and female urinary bladder; the third is on the mechanism of vomiting; the fourth gives an instructive case of aneurism of the femoral artery cured by ice. Our friend, Lewis A. Sayre, M. D., has an interesting case, in the next article, of abscess from pneumonia of the left lung, in a tuberculous patient, terminating favorably, by forming a fistulous opening between the third and fourth ribs, and an abscess in the substance of the lung. A preparation was exhibited by the President of the Society illustrative of a case in which there had been caries of the dorsal vetebræ, and the discharge had communicated with the right pleural cavity. It was associated with tuberculous disease, and the matter was discharged by an opening between the fifth and sixth ribs. As long as the opening remained, the expectoration was trifling, but on its closure, profuse expectoration took place, and when the opening was again made, the expectoration ceased. After a second closure of the opening, the patient died, apparently suffocated by the immense quantity of fluid with which the bronchiæ became filled. In the autopsical examination, it was discovered that the lung had almost entirely disappeared. An elaborate and learned paper follows, on carcinoma in general, and cancer of the stomach. The statistics of this paper are full, and of a highly interesting character. Various morbid specimens were presented to the society, among the rest, one of the cranium of a young woman who had died of secondary syphilis, the whole parietal bone of which was riddled, the outer table being nearly destroyed; one of a lateral curvature of the spine, in which the dorsal stood at a full right angle to the lumbar vertebræ; and the cervical again at a like angle to the dorsal; one of a laceration of the cerebellum of a woman who had fallen, striking on the back of her head; and one of a calculus composed of xanthic oxide, from the cabinet of Professor Parker, being the fifth of the kind on record. The President announces that circular letters will be sent to the members of the association, asking their attention to certain specific objects of inquiry. As an example, he proposes the following as a subject for the investigation of the members: "Have you known any form of fever to occur in the neighborhood of decomposing animal matter? If so, can it be fairly attributed to such decomposition, and what is the nature of such disease?"

